# Carboxylated superparamagnetic Fe_3_O_4_ nanoparticles modified with 3-amino propanol and their application in magnetic resonance tumor imaging

**DOI:** 10.1186/s12885-023-10514-0

**Published:** 2023-01-16

**Authors:** Changyuan Wang, Yang Wang, Wangchuan Xiao, Xiaohua Chen, Renfu Li, Zhiyong Shen, Fengchun Lu

**Affiliations:** 1 Department of Pediatric Surgery, Fujian Medical University Union Hospital, No. 29 Xinquan Road, 350001 Fuzhou, Fujian China; 2 School of resources and chemical engineering, Sanming University, No. 25, Jindong Road, 365004 Sanming, Fujian China; 3 Department of Pancreatic Surgery, Fujian Medical University Union Hospital, No. 29 Xinquan Road, 350001 Fuzhou, Fujian China

**Keywords:** 3-amino propanol, Nanoparticles, Magnetic resonance imaging, Tumor, Fe_3_O_4_

## Abstract

**Background:**

Ultrasmall superparamagnetic iron oxide (USPIO) nanoparticles are of potential magnetic resonance imaging (MRI) contrast agents for tumor diagnosis. However, ultrasmall particle size or negative surface charge lead to relative short half-life which limit the utilization of USPIO for in vivo MRI contrast agents.

**Methods:**

Superparamagnetic Fe_3_O_4_ nanoparticles coated with polyacrylic acid (PAA)were synthetized, and modified by 3-amino propanol and 3-diethyl amino propyl amine. The characteristics of superparamagnetic Fe_3_O_4_ nanoparticles were investigated through transmission electron microscopy, X-ray diffraction analysis, Zata potential analysis, thermogravimetric analysis, and relaxation properties analysis. Magnetic resonance imaging animal experiment was performed.

**Results:**

The synthetized nanoparticles were irregular spherical, with small particle size, few agglomeration, and good dispersion in water. After modification, the potential fluctuation of nanoparticles was small, and the isoelectric point of nanoparticles changed to high pH. After 3-amino propanol modification, the weight loss of the curve from 820 to 940 °C was attributed to the decomposition of 3-amino propanol molecules on the surface. The T1 relaxation rate of nanoparticles changed little before and after modification, which proved that the modification didn’t change the relaxation time. Brighter vascular images were observed after 3-amino propanol modification through measurement of magnetic resonance tumor imaging.

**Conclusion:**

These data indicated the Fe_3_O_4_ nanoparticles modified by 3-amino propanol should be a better contrast agent in the field of magnetic resonance tumor imaging.

## Introduction

Magnetic resonance imaging (MRI) is a noninvasive and radiation-free medical imaging technology, which has become one of the most effective clinical means to diagnose soft tissue lesions [1]. In the diagnosis of various tumor diseases, gadolinium contrast agents are often injected clinically to enhance the contrast of the target blood vessels [2]. However, gadolinium contrast agents have a short half-life in vivo and are prone to generate background noise, so the scanning time window in the equilibrium period is short [3]. In addition, gadolinium contrast agent has the risk of renal fibrosis [4].

Ultrasmall superparamagnetic iron oxide (USPIO) nanoparticles contrast agents have long half-life and widened acquisition time window [5]. In addition, USPIO can be used for high-resolution scanning in equilibrium period, which has high clinical application value. After injecting USPIO, multiple regions of interest could be scanned at the same time, such as angiography, local tumor imaging, and provincial metastasis imaging that requires respiratory gating and other imaging time [6]. As a supplement to the first pass imaging, the balance phase scan can more comprehensively show the metastasis of other parts of the body [7]. For tumor patients, after one injection, the patient can be repeatedly checked. Meanwhile tumors could be tracked and analyzed.

As a blood pool contrast agent, USPIO has the advantages of long half-life, biodegradability, and can be metabolized by cells into human normal plasma [8]. As T2 contrast agent, USPIO’s main strengthening mechanism is magnetic susceptibility effect, and its relaxation rate is about 7 ~ 10 times of Gd^3+^ under the same conditions [9]. A small dose can produce a local magnetic field gradient several orders of magnitude higher than that of paramagnetic contrast agent. USPIO uses the high permeability of malignant tumors to macromolecular contrast agents to differentiate benign and malignant tumors [10]. In normal tissues or benign tumors, USPIO could not pass through the complete capillary basement membrane, and there were no blue dye particles under the light microscope [11]. Moreover, tumor cells can phagocytize iron particles, so USPIO can improve the specificity of tumor diagnosis [12].

At present, the main preparation methods of USPIO are: co-precipitation method, high temperature thermal decomposition method, and polyol method. These three methods can regulate the particle size of USPIO, and each has its advantages and disadvantages. The co-precipitation method has mild conditions and is easy to be prepared on a large scale, but the monodispersity of the nanoparticles is poor [13]. The nanoparticles obtained by high-temperature thermal decomposition method have high crystallinity, narrow particle size distribution, and the accuracy of particle size regulation can be controlled within 1 nm [14]. However, the reaction needs to be carried out under high temperature conditions. The nanoparticles obtained are oil-soluble. Before being applied to biomedicine, additional phase transfer should be carried out to convert oil solubility to water solubility. The steps are complex, and the application is limited to a certain extent [15]. Polyol is a polar solvent, which can dissolve most inorganic metal salts to form a solution, and its high boiling point can provide high reaction temperature. It has strong reducibility at high temperature, and has been widely used to prepare nano-metal and oxide particles.

Polyethylene glycol is commonly used as surface coating agent [16]. The surface coating content and the size of nanoparticles are mutually restricted. The particle size is negatively correlated with surface organic matter content. Same size nanoparticles with different thickness of surface coating agent are hardly achieved [17]. In this study, polyacrylic acid coated nanoparticles were prepared first, and then EDC/NHS was used as a coupling agent to connect 3-amino propanol to convert the surface carboxyl groups of nanoparticles into hydroxyl groups. This ensures that the surface hydroxyl coated nanoparticles can be obtained without changing the particle size, which is conducive to extend half-life in vivo without changing the relaxation property. 3-amino propanol was used as an alkaline hydrolysate, which was a polar liquid and could be miscible with polyols. 3-amino propanol has both amino and hydroxyl groups, and the hydroxyl group is reducible. The amino group is alkaline, has a high boiling point (175 °C), and is miscible with ethylene glycol [18]. Therefore, this method is simple and easy to repeat.

In this study, USPIO with particle size 8–10 nm was successfully prepared with 3-amino propanol as hydrolysate. The application of 3-amino propanol modified USPIO as magnetic resonance blood pool contrast agent was investigated.

## Methods

### Synthesis of superparamagnetic Fe_3_O_4_ nanoparticles

Polyacrylic acid (0.3614 g) and anhydrous ferric chloride (0.3320 g) were put into a beaker containing 15.0 mL diethylene glycol solvent. The mix solutions were stirred with a glass rod, and heated at 200 °C. Sodium hydroxide solid (1 g) was dissolved in 10.0 mL diethylene glycol and dissolved by ultrasound and heated to 80 °C. 2 mL diethylene glycol solution containing sodium hydroxide were added to mix solutions. After 10 min, heating was stopped. Ethyl acetate was added and black precipitate was obtained. The upper clear liquid was poured after centrifuging, and the black precipitate was dissolved with absolute ethanol, and this step was repeated 4 times. The precipitate was dissolved with 10 mL distilled water, and lyophilized.

### Modification of superparamagnetic Fe_3_O_4_ nanoparticles by 3-amino propanol

Prepared nanoparticles (2 mL) were put into conical flask. 0.1153 g N-hydroxysuccinimide (NHS) were dissolved in 5 mL distilled water, and put into the conical flask. 0.161 g 3-dimethylaminopropyl-3-ethyl carbodiimide hydrochloride (EDC) were dissolved in 5 mL distilled water, and put into the conical flask. 130 µL 3-amino propanol were put into the conical flask. The mixed solutions were oscillated for 24 h, and EDC (0.0344 g) solution was added every 30 min during oscillation. After filtration, the solutions were lyophilized.

### Modification of superparamagnetic Fe_3_O_4_ nanoparticles by 3-diethyl amino propyl amine

Prepared nanoparticles (2 mL) were put into conical flask. 0.1116 g NHS were dissolved in 5 mL distilled water, and put into the conical flask. 0.171 g EDC were dissolved in 5 mL distilled water, and put into the conical flask. 200 µL 3-diethyl amino propyl amine were put into the conical flask. The mixed solutions were oscillated for 24 h, and EDC (0.0205 g) solution was added every 30 min during oscillation. After filtration, the solutions were lyophilized.

### Morphology analysis of nanosphere

The diluted nanoparticles were dropped onto the copper net sprayed with carbon film, covered with Petri dishes and dried naturally. The regular shape of magnetic nano-microspheres and their distribution in the aqueous dispersion were observed by transmission electron microscope.

### X-ray diffraction analysis

The lyophilized nanoparticles were used for X-ray diffraction analysis. The half peak width of the diffraction peak of magnetic nanoparticles were scanned by X-ray diffractometer (D/max-2200/PC, Rigaku, Japan), which indicated the composition and average grain size of nanoparticles.

### Particle size and Zata potential analysis

The nanoparticles were prepared as described in 2.1, 2.2, and 2.3. The particle size and Zata potential of each solution were measured by laser nano particle sizer (Malvern, UK).

### Thermogravimetric analysis

The nanoparticles were prepared as described in 2.1, 2.2, and 2.3. The content of organic matter on the surface of nano particles before and after modification was measured by thermogravimetric analyzer (SPA449F, Netzsch, Germany).

### Relaxation properties analysis

The nanoparticles solutions were adjusted to different concentration ranging from 0.01 to 5.5 mM. Then, nuclear magnetic resonance relaxation meter (Mq60, Bruker, Germany) was used to test the relaxation signal.

### Magnetic resonance imaging animal experiment

New Zealand white rabbits (2–3 months, 2.5-3 kg) purchased from Charles River (Beijing, China) were used in this study. Animals were divided into 3 groups including group Gadolinium butoxide, group Fe_3_O_4_ nanoparticles, and group Fe_3_O_4_ nanoparticles modified by 3-amino propanol. VX-2 tumor specimen was used for rabbit tumor implantation. After anaesthesia with ketamine (40 mg/kg) and xylazine (5 mg/kg) through intramuscular injection, the parts of hind limbs were shaved and disinfected. Minced VX-2 tumor (0.5 mL) were injected into the hind limbs of animals with 16-gauge needle. After 2 weeks, the tumors were around 1–2 cm, and magnetic resonance imaging was conducted. The animals were anaesthetized by injected ketamine (40 mg/kg) and xylazine (5 mg/kg) intramuscularly before magnetic resonance. Gadolinium butoxide (100 µmol/kg), Fe_3_O_4_ nanoparticles (40 µmol/kg), and Fe_3_O_4_ nanoparticles modified by 3-amino propanol (40 µmol/kg) were injected intravenously into animals in different groups. The parameters of magnetic resonance instrument were set as follows: TR = 2.79, TE = 1.14 ms, fov (region of interest): 160*200mm, matrix: 192*192, spatial resolution: 1 mm*1mm*1mm. Scan was performed once before injecting the contrast agent. 1 h after injection contrast agent, scanning was performed. The signal measurement area was located in the tumor area. The tumor intensity signal was measured at 6 fixed points of tumor in each animal, and average value was calculated.

### 10 CCK8 assay

The cytotoxicity after modification was measured with CCK8 method. Briefly,

Cells (2 × 10^4^/well) were seeded into 96-well plates. After 24 h, the cells were treated with different concentrations of superparamagnetic Fe_3_O_4_ nanoparticles (5, 10, 20, and 40 µmol/mL) for 24 h. Then, the CCK-8 kit (#C0037, Beyotime, Beijing, China) was used to detected cell proliferation according to the instruction.

## Results

### Identification of superparamagnetic Fe_3_O_4_ nanoparticles

**Fig. 1:**
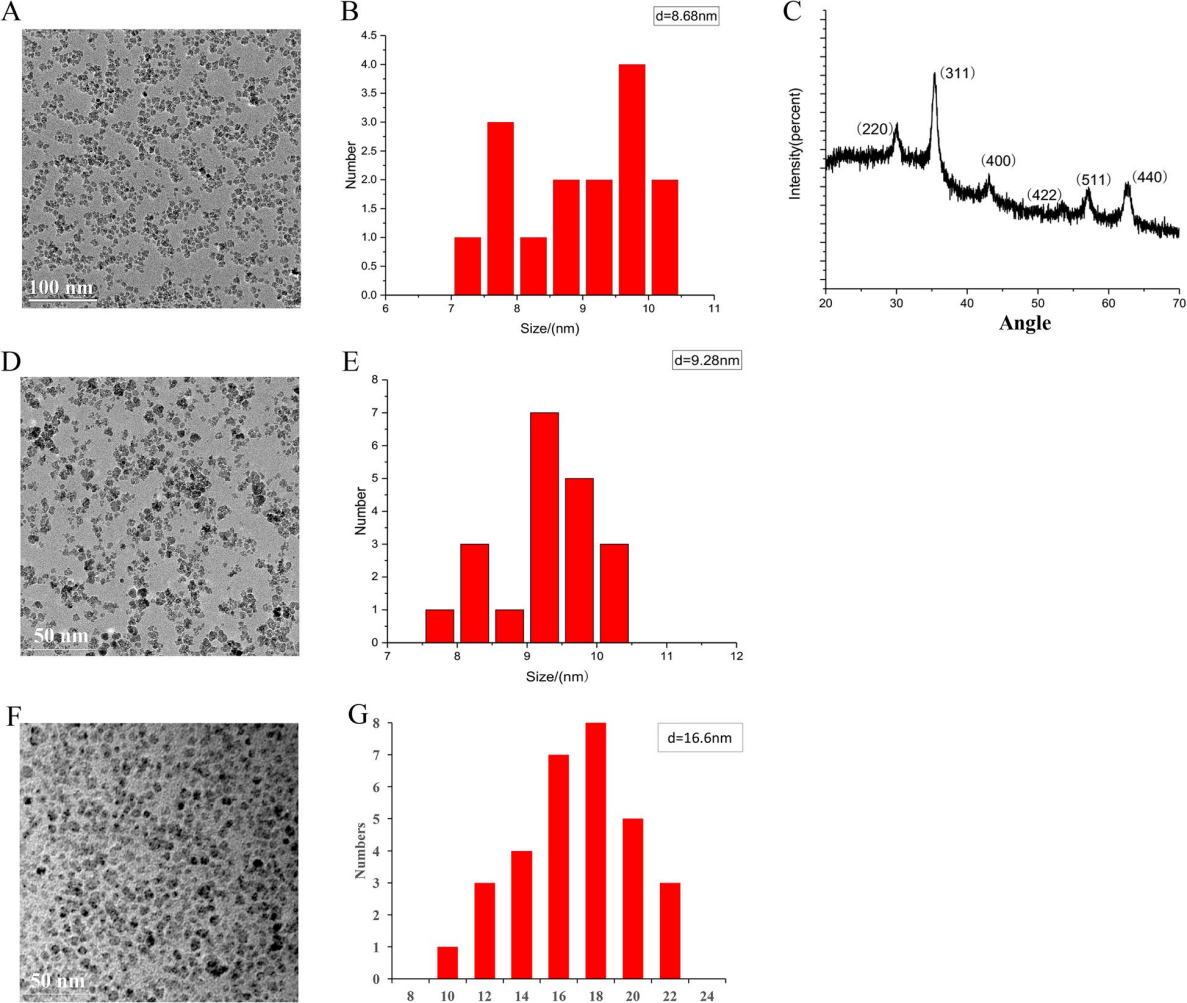
Synthesis and identification of superparamagnetic Fe_3_O_4_ nanoparticles. **A** High resolution transmission electron microscopy analysis of superparamagnetic Fe_3_O_4_ nanoparticles; **B** Average particle size of nanoparticles; **C** X-ray diffraction analysis; **D** High resolution transmission electron microscopy analysis of superparamagnetic Fe_3_O_4_ nanoparticles modified by 3-amino propanol; **E** Average particle size of nanoparticles; **F** High resolution transmission electron microscopy analysis of superparamagnetic Fe_3_O_4_ nanoparticles modified by 3-diethyl amino propyl amine; (**G**) Average particle size of nanoparticles

The synthetic superparamagnetic Fe_3_O_4_ nanoparticles was identified by transmission electron microscopy (Fig. 1A). We found that nanoparticles were irregular spherical, with small particle size, and few agglomeration. The average particle size of nanoparticles was 8–10 nm (Fig. 1B). The result of X-ray diffraction analysis indicated that six diffraction peaks in total were observed, and they were distributed at 30.20°, 36.55°, 43.27°, 54.58°, 57.43°, and 63.73° (Fig. 1C). The corresponding nanoparticles crystal planes are (220), (311), (400), (422), (511), (440), respectively. The sized nanoparticles after modification by 3-amino propanol or 3-diethyl amino propyl amine were also investigated through transmission electron microscopy. The average particle size of nanoparticles modified by 3-amino propanol was 9–10 nm (Fig. 1D-E). The average particle size of nanoparticles modified by 3-diethyl amino propyl amine was 16–20 nm (Fig. 1F-G).

#### Particle size distribution of nanoparticles analysis after surface modification by 3-amino propanol or 3-diethyl amino propyl amine

**Fig. 2:**
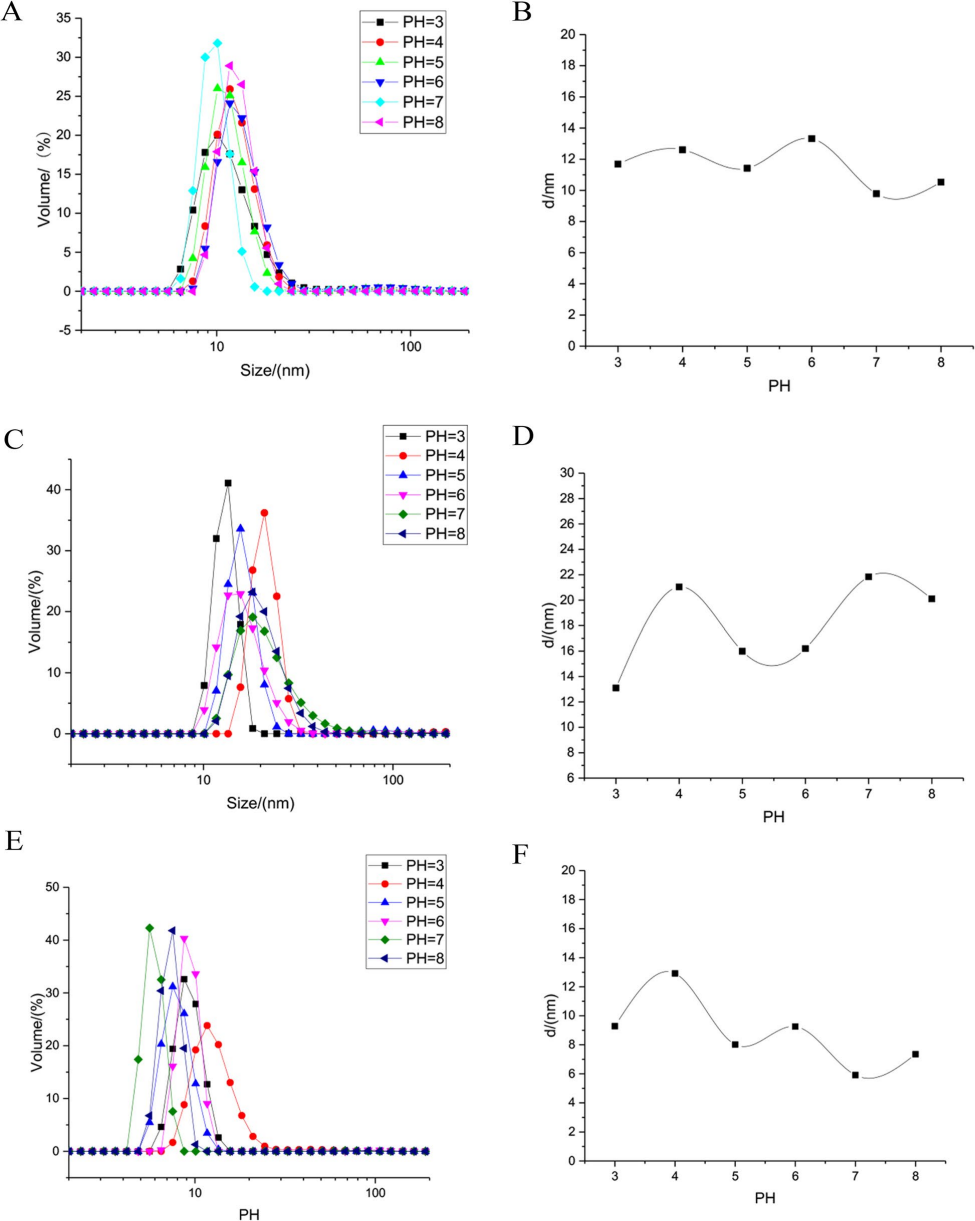
Particle size distribution detection after surface modification by 3-amino propanol or 3-diethyl amino propyl amine. **A** The particle size distribution was measured before surface modification; **B** The average hydrated particle size of nanoparticles with different pH before surface modification; **C** The particle size distribution was measured after surface modification by 3-amino propanol; **D** The average hydrated particle size of nanoparticles with different pH after surface modification by 3-amino propanol; **E** The particle size distribution was measured after surface modification by 3-diethyl amino propyl amine; **F** The average hydrated particle size of nanoparticles with different pH after surface modification by 3-diethyl amino propyl amine

Particle size distribution of nanoparticles analysis after surface modification by 3-amino propanol or 3-diethyl amino propyl amine was performed on the condition of different pH. The particle size distribution was measured before surface modification firstly (Fig. 2A). The size distribution of nanoparticles was between 7 and 11 nm, mainly between 8 and 10.5 nm (Fig. 2A). The average hydrated particle size of nanoparticles with different pH is between 9.97 and 13.1 nm (Fig. 2B). It can be concluded that the particle size range of nanoparticles prepared by polyols was relatively narrow, and the size of nanoparticles was relatively small.

After surface modification by 3-amino propanol, the size distribution of nanoparticles was between 4 and 25 nm, mainly between 7 and 10 nm (Fig. 2C). The average hydrated particle size of nanoparticles with different pH was between 6 and 13 nm, but it was almost 8 nm. (Fig. 2D). After modification by 3-amino propanol, the particle size distribution of nanoparticles was wider than that before modification, indicating that amino group partially replaced hydroxyl group in carboxyl group. The particle size of the nanoparticles after modification by 3-amino propanol was smaller than that before modification, indicating that the nanoparticles were slightly hydrolyzed. After surface modification by 3-diethyl amino propyl amine, the size distribution was around 9–30 nm, mainly from 15 to 25 nm (Fig. 2E). The average hydrated particle size of nanoparticles was between 13 and 23 nm, but it was almost 15 nm. (Fig. 2F).

### Zeta potential and thermogravimetric analysis after surface modification

**Fig. 3:**
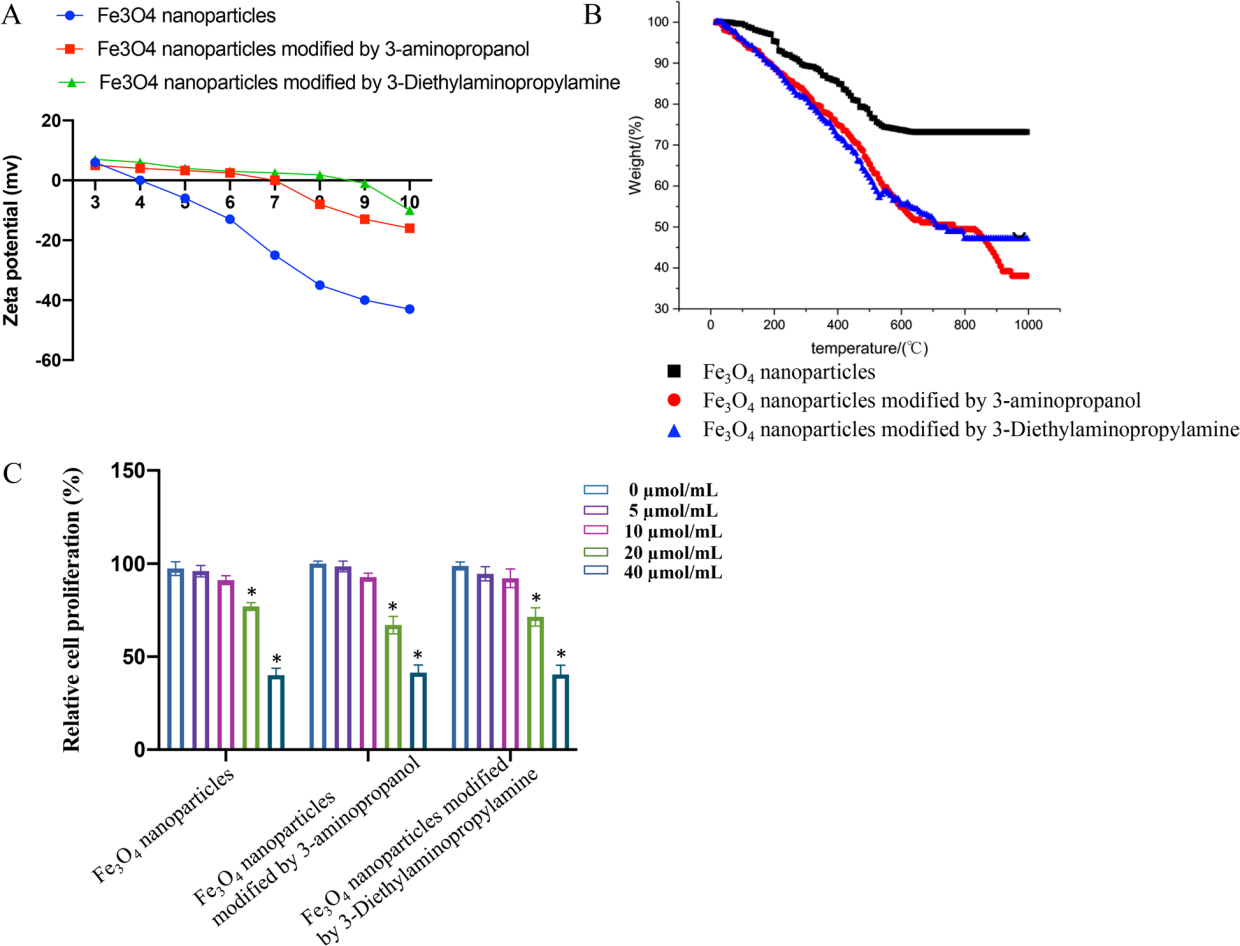
Zeta potential and thermogravimetric analysis after surface modification. **A** Zeta potential analysis after surface modification. **B** Thermogravimetric analysis after surface modification. **C** Cell proliferation was measured with CCK8 method (* indicates *p* < 0.05 compared with group 0 µmol/mL)

With the change of pH from 3 to 8, the isoelectric point of nanoparticles before modification was equal to 4 at pH, which indicated that Fe^3+^ existed in the solution. When pH was between 4 and 8 and the potential was less than zero, indicating that carboxyl groups were stable and free Fe^3+^ was hydrolyzed and precipitated (Fig. 3A). After modification, the potential fluctuation of nanoparticles was small. The potential of nanoparticles coated with 3-diethyl amino propyl amine was more positive than that of 3-amino propanol, because there were hydroxyl groups in 3-amino propanol. These indicated that the smaller the particle size of nanoparticles, the greater the potential, the better the dispersion in water. After modification, the isoelectric point of nanoparticles changed to 7 and 8, respectively.

Between room temperature to 200 °C, the weight loss of nanoparticles was due to the evaporation of free water and bound water. The weight loss of group Fe_3_O_4_ from 200 to 555 °C was mainly due to the pyrolysis of polyacrylic acid carbon chain on the surface of Fe_3_O_4_ nanoparticles, and the weight loss rate was 26.54% (Fig. 3B). After 3-amino propanol modification, the weight loss of the curve from 820 to 940 °C was attributed to the decomposition of 3-amino propanol molecules on the surface and the breaking of a small amount of unsubstituted polyacrylic acid molecules. The weight loss rate was 10.23%. After the modification of 3-diethyl amino propyl amine, the weight loss of the curve from 200 to 800 °C could be attributed to the decomposition of unreacted 3-diethyl amino propyl amine molecules and the breaking of polyacrylic acid molecules in nanoparticles, and the weight loss rate was about 39.48%.

The influence of modification on cell proliferation was investigated. Significant decrease of cell viability was observed after treatment with 20 or 40 µmol/mL Fe_3_O_4_ nanoparticles compared with group 0 µmol/mL (Fig. 3C). Similar findings were found after treatment with Fe_3_O_4_ nanoparticles modified by 3-amino propanol or 3-diethyl amino propyl amine (Fig. 3C). These results suggest that modification won’t exert negative effect on cytotoxicity.

### Relaxation properties analysis after surface modification

**Fig. 4:**
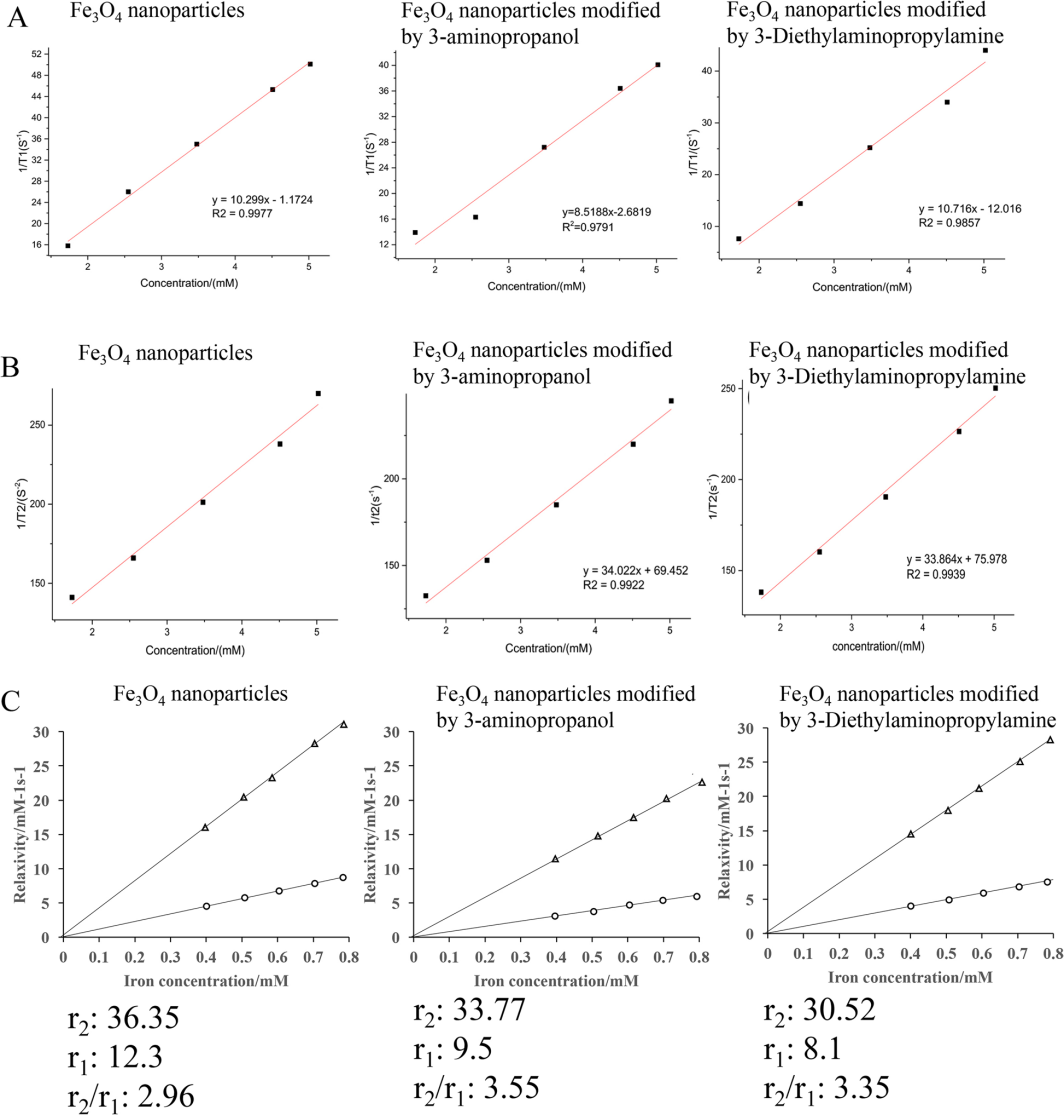
Relaxation properties analysis after surface modification. **A** The values of 1/T1 with change of Fe_3_O_4_ concentration were measured before and after modification; **B** The values of 1/T2 with change of Fe_3_O_4_ concentration were measured before and after modification; **C** The values of r_1_ and r_2_ were analyzed

The values of 1/T1 (Fig. 4A) and 1/T2 (Fig. 4B) with change of Fe_3_O_4_ concentration were measured before and after modification. We found that with the increase of Fe^3+^ concentration, the larger 1/T1, the smaller T1 and the higher relaxation efficiency. The T1 relaxation rate of nanoparticles changed little before and after modification, which proved that the modification didn’t change the relaxation time (Fig. 4A). Similar data were observed regarding 1/T2, and the weakened T2 signal intensity indicated that the T2 weighted image was dimmed (Fig. 4B). In addition, the value of r_2_/r_1_ was significantly increased after modification (Fig. 4C).

### Application of 3-amino propanol modified superparamagnetic Fe_3_O_4_ nanoparticles in magnetic resonance tumor imaging

**Fig. 5:**
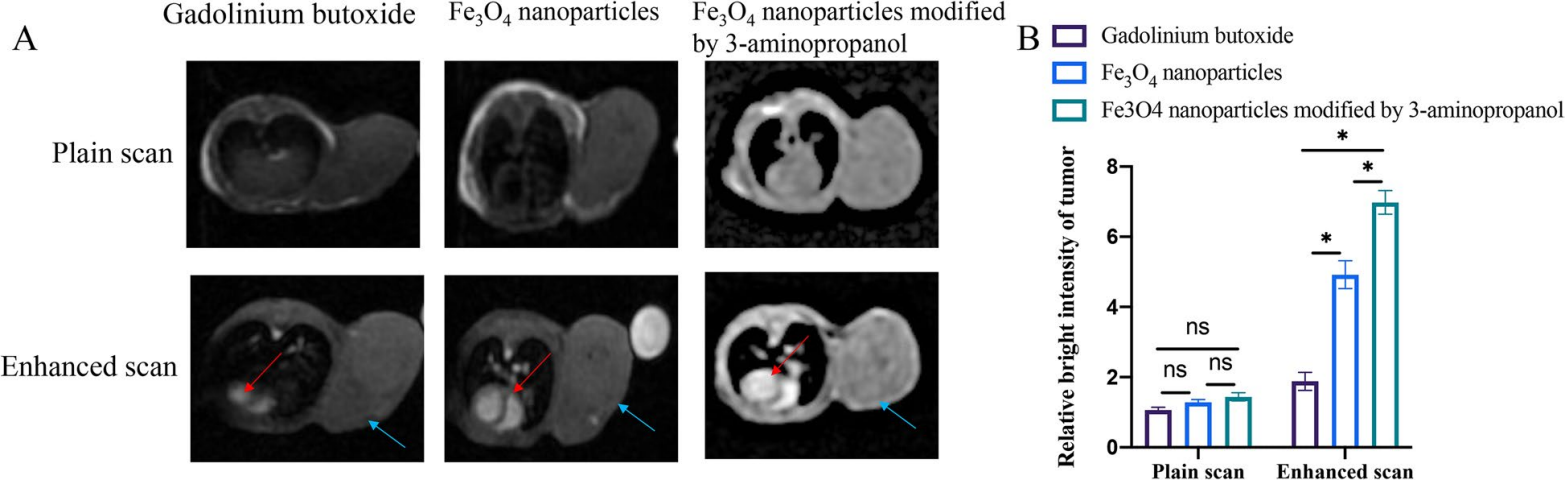
Application of 3-amino propanol modified superparamagnetic Fe_3_O_4_ nanoparticles in magnetic resonance tumor imaging. **A** Tumor imaging was investigated through MRI. **B** The tumor brightness was analyzed. (* indicates *p* < 0.05 compared with group Gadolinium butoxide; ns indicates no significant difference, red arrows indicate heart position, and blue arrows indicate tumor position)

1 h after contrast agent injection, tumor imaging was investigated through MRI. In the group Gadolinium butoxide, the target tumor image was blurred (Fig. 5A-B) due to the short half-life in vivo. However, in the group Fe_3_O_4_ nanoparticles, clear vessel image was observed. In addition, brighter vascular images were observed after 3-amino propanol modification. These data indicated the Fe_3_O_4_ nanoparticles modified by 3-amino propanol should be a better contrast agent in the field of magnetic resonance tumor imaging.

## Discussion

This study puts forward a new method for the preparation of superparamagnetic Fe_3_O_4_ microspheres. The superparamagnetic Fe_3_O_4_ nanoparticles were synthesized by using diethylene glycol as the hydrolysis agent, anhydrous FeCl_3_ as the iron source, polyacrylic acid (PAA) as the stabilizer of carboxyl group, and sodium hydroxide solid dissolved in diethylene glycol mixture as the alkaline hydrolysis agent. Diethylene glycol has stronger polarity than ethylene glycol, and it is easily soluble in organic matter as solvent, and has high boiling point, so it provides higher reaction temperature [19]. The proportion of diethylene glycol was increased, the polarity of the system was increased, and the particle size of microspheres was decreased [20]. The hydroxyl group in diethylene glycol has reducibility, so this material is used as a solvent to prepare superparamagnetic Fe_3_O_4_ nanoparticles.

In this study, the particle size of nanoparticles modified with 3-amino propanol or 3-diethy lamino propyl amine was larger than that before modification, and the Zata potential was close to zero. Thermogravimetric analysis and relaxation performance analysis were ideal. Preparation of USPIO by polyol method was chose, and polyol method has unique advantages, compared with co-precipitation method and high temperature thermal decomposition method. The iron precursor acetylacetone iron used in the high temperature thermal decomposition method can be directly decomposed into USPIO in polyols without heating any additives [21, 22]. Ferric chloride used in the coprecipitation method can also be dissolved in polyols, alkaline hydrolysate and protective agent are added, and USPIO can also be obtained by heating reaction [23]. USPIO with precise particle size control can be obtained from these two different precursors, and the obtained USPIO is water-soluble with good monodispersity [24]. The surface of USPIO with iron acetylacetonate as precursor is coated with polyols, which is not easy to be further modified [25]. With ferric chloride as precursor, small molecules or macromolecules containing carboxyl, amino and other compounds can be added to obtain USPIO with different surface coating, which is more widely used [26]. The thermal stability of materials is one of the indicators to select the performance of materials [27]. Thermogravimetric analysis can be carried out on samples to detect the relationship between sample quality and temperature [28]. Whether the sample will be chemically decomposed under high temperature conditions and the thermal stability of the sample can be measured by the mass loss on the TGA curve.

In this research, almost same cell proliferation ability was observed among different groups on the condition of different concentrations, which proved that the modification didn’t exert more cytotoxicity (Fig. 3C). The isoelectric point before modification was 4, which corresponds to the isoelectric point of carboxyl group. After modification, the isoelectric point was around 7 (Fig. 3A). The change of isoelectric point suggested that the carboxyl group was replaced by hydroxyl group. The surface was transformed from carboxyl group to hydroxyl group, and the thickness of surface organic matter increased, but the relaxation rate was not changed significantly, which is conducive to the subsequent application of magnetic resonance contrast agent.

Meanwhile, through in vivo tumor imaging experiment, we found that the 3-amino propanol modified superparamagnetic Fe3O4 nanoparticles presented a significant brighter vascular image compared with group Gadolinium butoxide and group Fe_3_O_4_ nanoparticles. This experiment indicates that 3-amino propanol modified superparamagnetic Fe3O4 nanoparticles have longer half-life in vivo. Considering the high safety of 3-amino propanol modified superparamagnetic Fe3O4 nanoparticles, it should be a good contrast agent in the field of magnetic resonance tumor imaging.

In this research, ultrafine superparamagnetic Fe_3_O_4_ nanoparticles were successfully synthesized with polyol as solvent, anhydrous FeCl_3_ as iron source, 3-amino propanol as alkaline hydrolysate and polyacrylic acid (PAA) as stabilizer. High resolution transmission electron microscopy, X-ray diffraction analysis and Raman spectroscopy confirmed that the synthesized nanoparticles were Fe_3_O_4_. Zeta potential and thermogravimetric analysis confirmed that the surface coated organic matter was polyacrylic acid. With the change of pH, the particle size does not change significantly (Fig. 2A-B), that is to say, water dispersion stability was good without agglomeration. In vivo magnetic resonance imaging experiments in rabbits suggested that the half-life of USPIO modified by 3-amino propanol in this study was longer than that of Gadolinium butoxide contrast agent used in clinic and Fe_3_O_4_ nanoparticles.

## Data Availability

The datasets used in the current study are available from the corresponding author on reasonable request.
